# Saliva as a non-invasive matrix for assessing xenobiotic metabolites and metabolomes: implications for maternal health and preeclampsia

**DOI:** 10.1038/s41368-025-00390-8

**Published:** 2025-07-22

**Authors:** Preethi Balan, Junfeng Zhang, Kok Hian Tan, Upul Cooray, Ryan WK Lee, Mah Lay Ong, Chaminda Jaya Seneviratne

**Affiliations:** 1 Singapore Oral Multiomics Initiative, National Dental Research Institute Singapore (NDRIS), National Dental Centre, Singapore, Singapore; 2 Oral Health ACP, Duke NUS Medical School, Singapore, Singapore; 3 Nicholas School of the Environment & Duke Global Health Institute, Duke University, Durham, NC USA; 4 Department of Maternal & Fetal Medicine, KK Women’s and Children’s Hospital, Singapore, Singapore; 5 Obstetrics & gynaecology ACP, Duke NUS Medical School, Singapore, Singapore; 6 Singapore Oral Population Health Initiative, National Dental Research Institute Singapore (NDRIS), National Dental Centre, Singapore, Singapore; 7 School of Chemistry and Molecular Biosciences, The University of Queensland, St Lucia, QLD Australia; 8 School of Dentistry, The University of Queensland, Brisbane, QLD Australia

**Keywords:** Predictive markers, Metabolomics

## Abstract

Chemical exposure during prenatal development has significant implications for both maternal and child health. Compared to blood, saliva is a non-invasive and less resource-intensive, alternative. Given the temporal variability of xenobiotic metabolites (XM), repeated sampling is essential. Therefore, saliva offers a valuable tool for the longitudinal assessment of prenatal exposomes. Despite its potential, no studies have explored saliva for XM measurement. This study pioneered using saliva to assess XM detectability and investigate the associations between prenatal XM and endogenous metabolomes in pregnant women. Saliva samples were analysed using mass spectrometry from 80 pregnant women at 24–34 weeks gestation. Metabolomes and exposomes were annotated using the Human Metabolome and U.S. Environmental Protection Agency databases. Metabolome-XM associations were clustered using Glay community clustering. Linear regression models, adjusted for age, estimated associations between catecholamines and XMs. XM levels were validated in a cohort of women (*n* = 14) with and without preeclampsia. Our study identified 582 metabolomes and 125 XM in saliva, demonstrating its potential as a matrix for exposure measurement. After false discovery rate correction, 18 109 significant metabolome-XM associations were identified. Community clustering revealed 37 connected clusters, with the largest cluster (238 nodes) enriched in tyrosine and catecholamine metabolism. Food-contact-chemicals and food-additives were significantly associated with higher catecholamine and their metabolite levels. Subgroup analyses revealed higher concentrations of these chemicals in women with preeclampsia compared to healthy controls. This study demonstrates that saliva contains valuable molecular data for measuring exposomes. Food-related chemicals were associated with higher catecholamine levels, which may be relevant to the prevalence of hypertensive crises in pregnancy.

## Introduction

The exposome encompasses the totality of all environmental exposures an individual encounters throughout their lifetime.^1^ Within this framework, xenobiotics refer to foreign compounds introduced into the body from external sources such as food, drinks, cosmetics, environmental pollutants, and pharmaceuticals.^2,3^ The concentration of such xenobiotic metabolites (XM) and the target organ dose an individual receives fluctuates over time, resulting in significant intra-individual variability.^4^ This variability introduces measurement errors, weakening causal associations and complicating disease risk assessments. Additionally, while some exposures are transient, their health effects may take weeks (e.g., peripheral blood effects), months (e.g., reduced bone marrow function), or even years (e.g., myelodysplastic syndromes or acute myeloid leukaemia) to become evident.^5^ As a result, serial monitoring of the xenobiotics over extended periods is necessary, but this can be resource-intensive when using blood. Saliva, however, offers a promising alternative to blood in exposure measurement, as it is a natural blood filtrate containing diverse molecular components that can provide valuable insights into an individual’s xenobiotic exposure. Bessonneau et al. using an open-source saliva-metabolome database identified 1 233 chemicals detectable in saliva, underscoring its potential as a rich source of molecular data for Exposome Wide Association Studies (EWAS).^6^ Despite its promise, saliva has been relatively underexplored as a matrix for comprehensive xenobiotic analysis, with only a few studies addressing this to date.^7,8^

During pregnancy, maternal exposure to XM may impact the foetus directly or by altering maternal metabolism and the intrauterine environment.^9^ Certain XMs can cross the placental barrier, enter foetal circulation, and disrupt development.^10^ The foetus is particularly vulnerable due to rapid organ growth, immature metabolism, and a higher relative exposure dose based on body weight.^11^ Evidence from the APrON cohort study demonstrated that exposure to phthalates, perfluoroalkyl acids, and non-essential metals is associated with pregnancy-induced hypertension.^12^ Hypertensive disorders during pregnancy increase the risk of adverse outcomes such as foetal growth restriction, placental abruption, preterm birth, and preeclampsia.^13^ Given the critical nature of these early life stages, any harmful exposure during this period warrants serious concerns and attention.

To address these research gaps, our study leverages saliva samples to examine the associations between prenatal chemical XMs and metabolomes. This approach allows for the exploration of molecular signatures and environmental exposures during pregnancy—a critical developmental period for maternal and foetal health—while circumventing the limitations of more invasive and difficult-to-collect specimens like blood.

## Results

### Profiling of salivary metabolomes and xenobiotic metabolites

Metabolomic profiling of the saliva samples identified 2 650 metabolites (positive and negative ion modes) after excluding mass ions with a relative standard deviation (RSD) > 30%. The identified metabolites were mapped to the HMDB, and 707 metabolites with confirmed human presence were retained after excluding predicted or expected metabolites. Based on HMDB database, these metabolites were classified into 321 endogenous metabolites, 125 exogenous metabolites, and 261 with both endogenous and exogenous origins. Although these 261 metabolites may stem from external exposures, their consistent synthesis within the body justifies their inclusion with endogenous metabolites for analytical clarity. Consequently, the dataset comprised 582 endogenous metabolites (termed the metabolomes) and 125 exogenous compounds (termed the xenobiotic metabolites (XM)) (Table S1).

Metabolome analysis using KEGG pathway database revealed its significant associations with carbohydrate metabolism (starch and glucose metabolism and the pentose phosphate pathway), amino acid metabolism (alanine, aspartate and glutamate, arginine, proline, tyrosine, histidine, glycine, serine and threonine metabolism), fatty acid metabolism (butanoate metabolism), nucleotide metabolism (purine metabolism), and detoxification process (glutathione metabolism) (Table 1, Fig S2).

**Table 1:** Pathways of metabolomes mapped to KEGG Pathway Database

Pathway Name	Match Status^a^	*P*-value^b^	FDR^c^	Impact^d^
Alanine, aspartate and glutamate metabolism	16/28	3.54E-8	1.69E-6	0.827
Arginine biosynthesis	11/14	4.24E-8	1.69E-6	0.598
Arginine and proline metabolism	17/36	5.20E-7	1.38E-5	0.698
Tyrosine metabolism	18/42	1.42E-6	2.84E-5	0.679
Histidine metabolism	8/16	4.05E-4	0.006	0.450
Glycine, serine and threonine metabolism	12/33	5.73E-4	0.007	0.618
Glutathione metabolism	10/28	0.001	0.015	0.236
Starch and sucrose metabolism	8/18	0.001	0.012	0.789
Pentose phosphate pathway	9/23	0.001	0.014	0.314
Butanoate metabolism	7/15	0.001	0.014	0.063
Purine metabolism	18/70	0.002	0.021	0.276

^a^Number of metabolites involved in the pathway

^b^The Fisher’s exact test was used to determine the association between salivary metabolites and pathways

^c^p-value corrected for False Discovery Rate (FDR)

^d^Impact represents the centrality of salivary metabolites in their respective pathways on a scale from 0 to 1

**Fig. 1:**
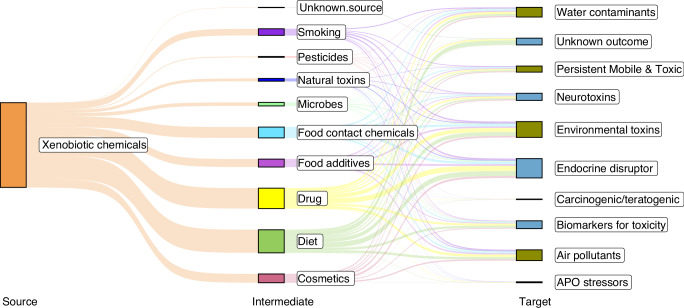
The Sankey diagram depicts the proportional relationships between the xenobiotic metabolite and its sources and effects. Each source of the xenobiotic metabolite is linked to potential effects and is colour-coded depending on the source. The width of the connecting lines indicates the relative strength of a particular source’s contribution to the xenobiotic metabolite or the effect

XMs were cross-referenced to various databases and chemical inventories to elucidate their potential origins and impacts on human physiological systems. Comprehensive details of the databases and resources utilised are provided in Table S2. Figure 1 shows the proportional distribution of the XM by potential sources and effects on human health and the environment. Sources of the XM included diet, drugs, food additives, pesticides, food-packaging plastics, natural toxins, and microbes. Additional sources included smoking and dermal absorption via cosmetic ingredients. The XM effects we identified included endocrine disruptors, neurotoxins, carcinogens, teratogens, and adverse pathway stressors (APOs), with many recognised as biomarkers of toxicity. A few identified compounds are among Persistent Mobile Toxic (PMT) substances that undergo extremely slow biodegradation in the environment, contributing to environmental (e.g., water, air) pollution.

### Network clustering of metabolomes and xenobiotic metabolites

**Fig. 2:**
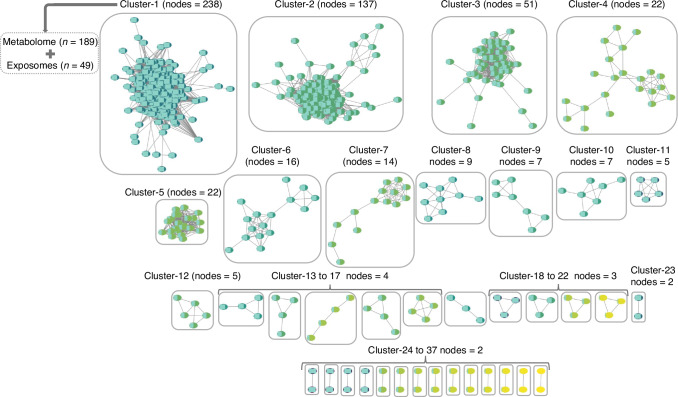
Network visualisation of endogenous-xenobiotic metabolite associations. The network displays 37 connected clusters identified using the Community Clustering Algorithm. Each cluster is labelled with its corresponding cluster number and the number of nodes it contains, arranged in descending order of node count

**Fig. 3:**
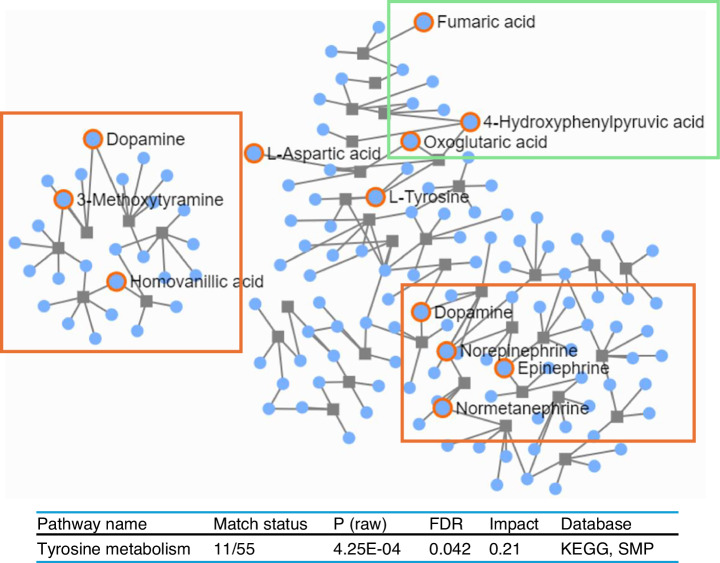
Visualisation of the tyrosine metabolism pathway. **a** The pathway view illustrates tyrosine metabolism, with significantly enriched metabolites in the dataset indicated by highlighted circles. Metabolites enclosed within the green box are involved in tyrosine catabolism, whereas those within the red box represent catecholamines and their metabolites. **b** The accompanying table provides a summary of the statistical analysis of the tyrosine pathway. The analysis was performed using MetaboAnalyst 6.0

Pearson correlation analysis, after FDR correction, identified 18,109 statistically significant associations between the metabolomes and XMs (Table S3, Fig. S3). We leveraged these associations to construct a metabolic network which comprised 598 nodes representing metabolites and 15 372 edges representing the correlation coefficient, with an average of 84.85 neighbours per node and a clustering coefficient of 0.75 (Table 2). Community clustering with the GLay algorithm classified the network into 37 connected clusters of varying sizes (Fig. 2). Among the 37 clusters identified, the largest cluster contained 238 nodes, constituting ~40% of the entire network. This cluster comprised 189 metabolomes and 49 XMs. The node attributes for this cluster are summarised in Table S3. The 189 metabolomes within this cluster, when mapped to the Small Molecule Pathway Database (SMPDB), revealed statistically significant enrichment in the tyrosine metabolism pathway (*P* = 0.04) (Fig. 3). This pathway involved 11 metabolites, of which 4-hydroxyphenyl pyruvic acid, oxoglutaric acid, and fumaric acid were involved in tyrosine catabolism, tyrosine and L-aspartic acid were essential amino acids and the remaining were catecholamines including dopamine, norepinephrine, and epinephrine, and their metabolites normetanephrine, 3-methoxy tyramine, and homovanillic acid.

**Fig. 4:**
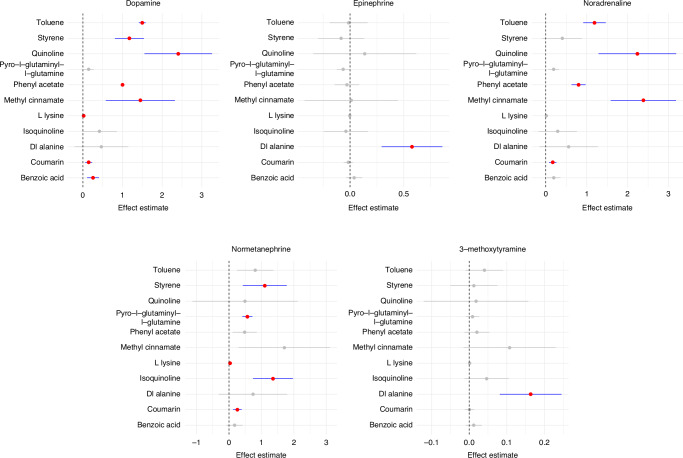
Forest plots depicting the associations between xenobiotic metabolite (y-axis) and catecholamines and their metabolites. The red dots represent the effect estimates, while the horizontal lines denote the 95% confidence intervals. Blue lines indicate statistically significant associations, while grey lines represent non-significant associations

**Table 2:** Network statistics summary

Network feature	Value
Number of nodes	598
Number of edges	15 372
Connected components	37
Average number of neighbours	84.85
Network diameter	5
Network radius	3
Characteristic path length	1.76
Clustering coefficient	0.75
Network density	0.35
Network heterogeneity	0.60
Network centralisation	0.42

Tyrosine metabolism links to the TCA cycle via intermediates like L-aspartic acid, oxoglutaric acid, and fumaric acid, primarily supporting energy metabolism rather than environmental exposure effects. Therefore, these metabolites were excluded, and only three catecholamines and their three metabolites were analysed further. Of the 49 XMs identified in the cluster, we selected 20 for further analysis based on their annotation in the Blood Exposome Database, a resource developed using U.S. EPA data. Focusing on these validated XMs enhanced the robustness and reliability of our findings. The remaining 36 clusters of metabolites did not exhibit significant pathway enrichment and were excluded from further analysis.

### Associations between catecholamines and xenobiotic metabolites

We performed a multivariate linear regression analysis using six catecholamine-associated variables as dependent variables and 17 XMs as independent variables, including biological age, gestational age, ethnicity, BMI, smoking status, BOP, and drugs (Allopurinol, Amlodipine, Fluoxetine, and Pseudoephedrine) as covariates.

The analysis revealed significant associations between 11 XMs and catecholamines (Fig. 4).

Dopamine levels were significantly associated with food-contact chemicals (FCCs), including toluene, styrene, and quinoline. Toluene and quinoline were also associated with noradrenaline levels, while styrene and isoquinoline demonstrated associations with normetanephrine.

The food preservative benzoic acid showed a significant association with dopamine levels. The food flavourant Dl-alanine was associated with both epinephrine and 3-methoxytyramine concentrations. In addition, the food additive coumarin exhibited associations with dopamine, noradrenaline, and normetanephrine. Methyl cinnamate was associated with both dopamine and noradrenaline, whereas L-lysine was associated with dopamine and normetanephrine.

### Determination of xenobiotic metabolite level in preeclampsia

**Fig. 5:**
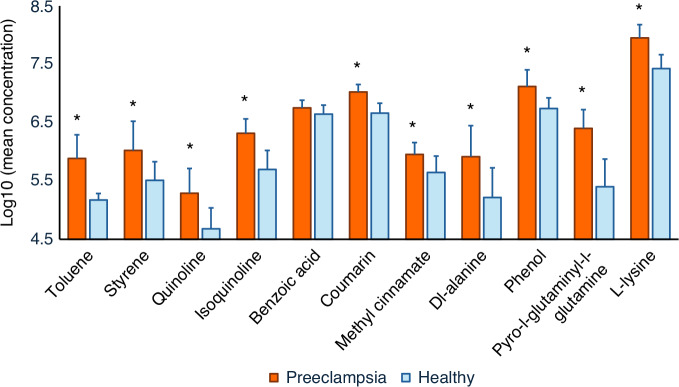
Comparison of Xenobiotic Metabolite Levels Between Pre-eclampsia and Healthy Groups. The bar graph displays the log10-transformed mean xenobiotic metabolite levels in women with pre-eclampsia (*n* = 7) and healthy controls (*n* = 7). Error bars represent the standard deviation of the mean. Significantly higher xenobiotic metabolite levels in the pre-eclampsia group than the control group are marked with an asterisk (*), indicating a *p* < 0.05

Given that catecholamines augment sympathetic tone and elevate blood pressure, we compared 11 XMs, significantly associated with catecholamines, in 7 women with pre-eclampsia and 7 age—and gestational-age-matched healthy controls (Table S4) in a case-control comparison within a cross-sectional study framework. Our analysis revealed that FCCs (toluene, styrene, isoquinoline and quinoline) and FAs (coumarin, phenyl acetate, DL-alanine, L-lysine, methyl cinnamate, and pyro-L-glutaminyl-L-glutamine) were present at significantly higher concentrations in women with pre-eclampsia compared to the healthy controls (Fig. 5).

## Discussion

This study, employing an untargeted metabolomic analysis, detected the presence of metabolomes and XMs in saliva, highlighting its potential as a valuable matrix for XM measurement. The study also showed significant associations between food-related chemicals, catecholamine levels, and preeclampsia, providing novel insights into the influence of the xenobiotics on metabolic pathways during the critical gestational period.

The human metabolome refers to the comprehensive set of metabolites with a molecular weight below 2 000 Da and includes those metabolites that humans ingest, metabolise, break down, or are exposed to refs. ^14,15^ They can originate endogenously through synthesis by enzymes encoded in our genome or microbial genomes or be exogenously introduced through food, drugs, and contaminants. Salivary glands are highly vascularised, allowing these blood-derived molecules to enter through transcellular and paracellular routes, affecting the biochemical composition of saliva.^16^ Accordingly, this study identified 582 metabolomes and 125 XMs in saliva. The identified metabolomes were linked to several critical biochemical pathways essential for energy production and redox balance.^17^ XM mapping revealed that the exogenously derived compounds predominantly originated from dietary sources. Consistent with our findings, Bessonneau et al. identified salivary molecules from various sources, including endogenous host and microbial metabolites, food, drugs, pollutants, and metals.^18^ This suggests that saliva, like other biological fluids, can detect metabolomes and XMs. We mapped our data against validated and peer-reviewed databases for both metabolomes and exposomes to ensure accurate identification and robust analysis of our salivary metabolites. Specifically, we utilised the HMDB, which has undergone extensive peer review by the metabolomics community and the data within the HMDB adheres to rigorous standards required for publication in peer-reviewed scientific journals and international conferences, ensuring its reliability. Additionally, we cross-referenced our findings with the U.S. EPA database, which maintains a comprehensive list of regulated chemicals under laws such as the Toxic Substances Control Act (TSCA). These lists aggregate chemicals associated with various scientific projects and publications, further enhancing the accuracy and relevance of our XM analysis.

The investigation into associations between metabolomes and XMs revealed 18 109 statistically significant interactions. The network constructed from these associations exhibited a high clustering coefficient of 0.75, indicating strong interconnectedness and suggesting potential biological interactions between the XM and metabolome. Given the scale and complexity of the biological interaction data, systematically exploring and visualising these large datasets posed a significant challenge. To address this, we decomposed the interaction network into communities of densely interacting nodes to infer functional modules using the Cytoscape GLay plugin, which employs layout algorithms optimised for large networks.^19^ A similar clustering approach has been employed in a multi-centre cohort study to identify signatures of the human early-life exposome.^20^ The metabolomes within the largest cluster were significantly enriched in tyrosine metabolism, with catecholamines (dopamine, norepinephrine, and epinephrine) and their metabolites (normetanephrine, 3-methoxytyramine, and homovanillic acid) being particularly relevant during pregnancy.^21,22^ Dopamine is secreted into the bloodstream and hydrolysed to norepinephrine and subsequently into epinephrine.^23^ Dopamine is also catalysed into methoxy tyramine and homo-vanillic acid, while norepinephrine and epinephrine are converted into normetanephrine and metanephrine, respectively. Dopamine, the “feel-good” neurotransmitter, is vital in the brain’s reward system, while epinephrine and norepinephrine are key to the sympathetic nervous system’s “fight or flight” response.^24^ Although these rapid responses are essential for survival, prolonged elevation of circulating catecholamines can lead to hypertension, cardiac disorders and motor dysfunction.^25,26^

FCCs are chemicals used in food packaging, storage containers, processing equipment, and kitchenware that can migrate into food and are subsequently get ingested.^27^ Our study found that solvents in FCCs—like toluene, styrene, quinolines, and isoquinoline—were significantly associated with most catecholamines and their metabolites. Previous studies show high toluene exposure (500 × 10^-6^–1 000 × 10^-6^) increases norepinephrine and dopamine in the brain,^28,29^ while toluene abuse during pregnancy can cause intrauterine growth retardation, premature delivery, congenital malformations, and postnatal developmental delays.^30^ Monoamine oxidase (MAO) is the key enzyme degrading norepinephrine, epinephrine, and dopamine. It exists in two isoforms: MAO-A, which deaminates norepinephrine and epinephrine, while MAO-B selectively deaminates dopamine. MAO inhibitors (MAOIs) block these enzymes, accumulating catecholamines.^31^ Studies have shown that styrene exposure decreases platelet MAO-B activity, possibly inducing catecholamine accumulation.^32^ Similarly, quinoline inhibits MAO-A, while isoquinoline, a structurally related compound, noncompetitively inhibits both MAO-A and MAO-B activity.^33^ Coumarin, used as an adulterant in commercial vanilla flavouring, is also an inhibitor of MAO.^34^ Although MAO-A inhibitors hold potential therapeutic value for psychiatric and neurodegenerative disorders, long-term administration of MOA inhibitors alters the firing rate and pattern of dopamine neurons.^35^ MAOIs are generally not recommended during pregnancy due to association with hypertensive crisis and foetal growth restriction.^36^

FAs, commonly used in processed foods for palatability, trigger dopamine release in the striatum, with pleasure proportional to dopamine levels.^37^ In this study, FAs such as methyl cinnamate, benzoic acid, and pyro-l-glutaminyl-l-glutamine were positively associated with dopamine levels, suggesting a similar mechanism of action. Methyl cinnamate (known for its fruity flavour) is metabolised into cinnamic acid and protects Tyrosine hydroxylase (TH) positive dopaminergic neurons, while sodium benzoate (preservative) stimulates TH expression, enhancing dopamine production.^38–40^ Pyroglutamyl peptides such as pyro-l-glutaminyl-l-glutamine formed during food processing reach the brain in sufficient concentrations and evoke dopamine release through glutamate-like activity.^41,42^ As dopamine increases, it is either recycled or metabolised into norepinephrine and epinephrine, aligning with the observed rise in their levels.^43^

In healthy pregnant women, plasma and urinary catecholamine levels remain unchanged.^44^ However, elevated catecholamines increase sympathetic adrenal tone, contributing to hypertension and platelet activation in pre-eclampsia.^45^ Women with pre-eclampsia show significantly elevated levels of catecholamines compared to normotensive pregnant women.^45–47^ Consistent with this, our study found that FCCs and FAs associated with elevated catecholamine were notably higher in pre-eclampsia. It should be noted that the small sample size limits the generalisability of the results. However, despite a small sample size, the relatively large magnitude of difference between the groups indicates a strong, biologically relevant effect, suggesting that food-related exposures may disrupt catecholamine levels, increasing the risk of hypertensive disorders and other maternal-foetal complications. Well-powered and covariate-controlled studies are needed for validation and generalisability.

A key limitation of this study is the dual origin of specific metabolites detected in biological samples. While databases like HMDB categorise metabolites as endogenous or exogenous, some metabolites classified as exogenous in this study have also been reported to be produced endogenously. For instance, phenylacetate, a flavouring agent (FEMA No. 3958), is also the metabolic end-product of phenylalanine (precursor to catecholamines).^48^ Similarly, L-lysine, a flavouring agent (FEMA 3847), is an essential amino acid obtained from dietary sources. This overlap complicates data interpretation, emphasising the need for further refinement of databases and accounting for dietary exposures to classify xenobiotics accurately and assess their impact on health outcomes. It also underscores the importance of accounting for socioeconomic status (SES) and physical activity in future studies, as these factors influence dietary patterns, environmental exposures, and metabolic processes relevant to maternal health and pre-eclampsia. Unfortunately, such data were not collected in the present study and are acknowledged as a limitation. Another limitation is the unknown threshold levels at which the identified XMs in saliva pose risks to pregnant women and the foetus. The XMs and the metabolomes in the saliva were measured only at one point per person, providing only a snapshot of the prenatal exposome-metabolomic responses. This cross-sectional study approach limits our ability to observe temporal changes or fluctuations in these biological markers and may not fully reflect long-term patterns or causal relationships. This limits the reproducibility and generalisability of the results to broader populations. Finally, validating metabolite levels using additional molecular assays, such as ELISA or Luminex, in the preeclampsia cohort would enhance the reproducibility and rigour of our results. Complementary molecular assays of relevance to the exposome present a valuable avenue for this line of research.

Nevertheless, this study examines explicitly a small but informative subset of the external chemical exposome detectable in human saliva. Saliva can effectively capture metabolomes and XMs, offering a non-invasive approach to monitoring chemical exposures. Participants are more inclined to donate saliva samples than venous blood, and the ability of subjects to self-collect saliva can enhance participation rates in EWAS studies, thereby generating larger biospecimen datasets. Repeated omics measurements across the lifespan can reduce exposure errors, strengthening the identification of chronic disease risk factors. Furthermore, this study highlights how the prenatal XM influences maternal catecholamine metabolism, potentially impacting birth outcomes like preeclampsia. As exposure to FCCs and non-nutritional FAs is preventable, raising awareness among scientists, policymakers, and industry leaders is essential to protect maternal and foetal health.

## Materials and methods

### Subject characteristics and biospecimens

In this cross-sectional study, 80 pregnant women between 24 and 34 weeks of gestation were recruited from KK Women’s and Children’s Hospital between October 2021 and December 2022. Eligibility criteria included participants being over 21 years of age and free from any pre-existing systemic conditions, including type 1 or type 2 diabetes mellitus, hypertension, cardiovascular, pulmonary, or renal disorders, as well as negative serological status for human immunodeficiency virus (HIV). Demographic data, anthropometric parameters, and medical conditions, including the diagnosis of pre-eclampsia, were obtained from hospital medical records. BMI was recorded during the first prenatal visit in the first trimester, and the oral glucose tolerance test (OGTT) was conducted between 24 and 28 weeks of gestation, per standard clinical guidelines for routine screening of gestational diabetes mellitus (GDM). All participants provided written informed consent before being included in the study. The study protocol was approved by the SingHealth Centralized Institutional Review Board (CIRB Ref No. 2020/2698).

We used saliva samples for this study based on the following considerations. (1) Like blood, saliva is in equilibrium with body tissues and can serve as a dynamic reservoir of small molecules that reflect the body’s physiological state at a given time.^6^ (2) Saliva collection is non-invasive and can be performed more readily than blood. With established protocols, saliva can be self-collected painlessly by study subjects. In this cohort of pregnant women, unstimulated saliva samples were collected from the subjects by the spit technique^49^ following a 60 min abstention from eating, drinking, or performing oral hygiene practices. This precaution was taken to ensure that the saliva samples minimise potential in situ (non-steady state) influences of dietary and synthetic compounds and oral microbes on the metabolome and the chemical XM. All samples were collected between 9:00 AM and 12:00 PM to reduce the impact of diurnal variation and were subsequently stored at –80 °C until further analysis. The subject characteristics are provided in Table 3.

**Table 3:** Subject characteristics (*n* = 80)

Maternal characteristics	Mean ± SD or *N* (%)
Age/years	31.1 ± 4.2
Gestation age/weeks	27.5 ± 3.2
Ethnic group	
Chinese	38.0 (47.5%)
Malay	25 (31.3%)
Indian	5 (6.3%)
others	12 (15%)
BMI	27.2 ± 4.5
Smoking history	
Current smoker	3 (3.8%)
Previous smoker	9 (11.3%)
None	68 (85%)
Oral glucose tolerance test/(mmol/L)	
Fasting	4.7 ± 0.8
1-hour	9.1 ± 2.6
2-hour	7.7 ± 2.2
Diastolic blood pressure/(mm Hg)	64.6 ± 10.7
Systolic Blood Pressure/(mm Hg)	112.0 ± 14.8
Pre-eclampsia	
Yes	7 (8.7%)
No	73 (91.2%)
Number of teeth present	27.9 (1.8)
Periodontitis diagnosis	
Yes	27 (33.8%)
No	53 (66.3%)
Periodontal pocket depth	2.2 ± 0.2
Calculated attachment loss	2.2 ± 0.2
Bleeding on Probing	26.4 ± 14.1
Mean Plaque score	38.4 ± 12.9

### Oral health measurements

Oral health measurements were taken for all teeth (excluding third molars) by a single examiner. Periodontitis was diagnosed based on interdental clinical attachment level (CAL) ≥ 2 mm at ≥2 nonadjacent teeth or buccal/lingual/palatal CAL ≥ 3 mm with pocket depth >3 mm at ≥2 teeth, following the 2017 World Workshop Classification of Periodontitis.^50^ Bleeding on Probing (BOP) and Plaque present were recorded on six sites per tooth (three buccal and three oral sites, respectively). Data were reported as percentages of total sites examined.

### Untargeted metabolomic analysis of saliva

Saliva samples were first centrifuged at 10 000 rpm for 10 min at 4 °C to obtain the supernatant, which was then mixed with cold methanol and acetonitrile. The mixture was subjected to high-speed centrifugation 14 000 × *g*, 10 min, 4 °C to precipitate proteins. The resulting supernatant, containing the extracted metabolites, was collected and used for subsequent analysis. Molecular separation and detection were conducted using a Waters UPLC I-Class Plus (Waters, USA) with a Q Exactive high-resolution tandem mass spectrometer (UPLC-Q Exactive MS) (Thermo Fisher Scientific, USA). A 5 µL aliquot of extracted saliva was injected into a Waters Acquity UPLC BEH C18 column (1.7 μm, 2.1 mm × 100 mm; Waters, USA) maintained at 45 °C. To ensure system stability, a pooled quality control (QC) sample was initially injected five times and subsequently every 10 samples during saliva analysis. The mobile phase for chromatographic separation consisted of 0.1% formic acid and acetonitrile for positive ionisation, while negative ionisation used 10 mmol/L ammonium formate and acetonitrile. The gradient elution conditions were as follows: 0–1 min at 2% B; 1–9 min from 2% to 98% B; 9–12 min at 98% B; 12–12.1 min returning to 2% B; and 12.1–15 min at 2% B, with a flow rate of 0.35 mL/min. Normalised collision energies were set at 20, 40, and 60 eV. ESI parameters included a sheath gas flow rate of 40×, auxiliary gas flow rate of 10×, and a spray voltage of 3.80 kV (positive) or 3.20 kV (negative). The capillary temperature was maintained at 320 °C, with an auxiliary gas heater temperature of 350 °C. Mass spectrometry data were acquired in full-scan mode (m/z range 70–1 050) at a resolution of 70 000, with an automatic gain control (AGC) target of 3e6 and a maximum ion injection time of 100 ms. Sample preparation, instrument settings, and data processing were conducted at BGI (Hong Kong, China).

### Ion peak extraction and identification

Raw chromatographic/mass spectrometric data were processed using Compound Discoverer 3.2 (Thermo Fisher Scientific, USA) for ion peak extraction, alignment, and integration. Parent ion mass deviation was set at <5 ppm, fragment ion mass deviation at <10 ppm, and retention time deviation at <0.2 min. Identified compounds were searched against several databases, including bmdb, ChemSpider™, mzCloud, HMDB, and PubChem. The resulting data matrix, containing chromatograph peak areas and identification details, was processed in MetaX. Data normalisation was performed using probabilistic quotient normalisation, and robust loess signal correction (R-LSC) was applied to adjust for batch effects. Compounds with a coefficient of variation exceeding 30% in QC samples were excluded. We used the K-Nearest Neighbors (KNN) method to impute missing values, and outlier detection was performed using a box plot based on the interquartile range (IQR) rule (Fig. S1).

### Statistical analysis

**Fig. 6:**
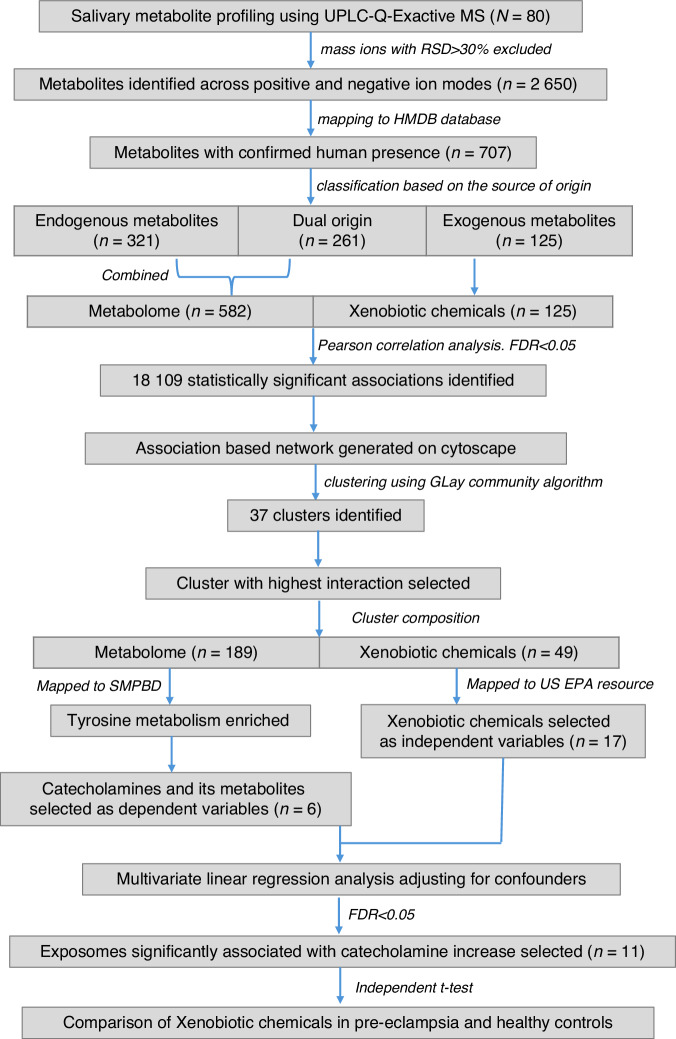
Metabolite analysis workflow. The figure shows the filtering, mapping and association of 582 metabolomes and 125 xenobiotic metabolites from saliva samples of 80 pregnant women

All statistical analyses were performed using R software (version 4.3.2; R Core Team, Vienna, Austria). The metabolites selected for analysis were filtered and classified based on their endogenous or exogenous origin using the Human Metabolome Database (HMDB).^51^ Pathway mapping was conducted using the Kyoto Encyclopedia of Genes and Genomes (KEGG) and the Small Molecule Pathway Database (SMPDB) using the Metaboanalyst 6.0 pipeline.^52^ Pathway enrichment p-values were calculated using the Hypergeometric Test, while pathway impact values were determined via pathway topology analysis by applying the Relative-Betweenness Centrality as the topology measure. Pearson correlation analysis was performed in R on log-transformed data with a threshold of correlation coefficient *r* > 0.5 and a statistical significance *P* < 0.05, adjusted for multiple comparisons via the False Discovery Rate (FDR) method using the p.adjust function in R. This network focused on metabolite-metabolite interactions without adjustments for confounders which were included in regression models. The metabolic/XM networks were constructed in Cytoscape (version 3.10.0), where individual compounds were represented as nodes and correlation coefficients were used as weighted edges to denote the strength of the associations between metabolites. Subsequently, network clustering was performed using the GLay community detection algorithm. Linear regression analyses were applied to the metabolites within the largest cluster, with FDR correction, adjusting for potential confounders including age, gestational age, ethnicity, BMI, smoking status, and BOP. In addition, drug metabolites such as Allopurinol, Amlodipine, Fluoxetine, and Pseudoephedrine—identified within the cluster and potentially influencing catecholamine levels—were also included as covariates in the adjustment.^53–55^ The validation of XMs included as independent variables in the regression models was conducted using the Blood Exposome List from the U.S. Environmental Protection Agency (EPA) resources.^56^ Independent t-tests were used to determine the differences in XM levels between women with pre-eclampsia and healthy controls. Figure 6 depicts the detailed workflow of metabolite analysis.

## Supplementary information

Supplemental material

Supplementary tables

## Data Availability

The data that support the findings of this study are available from the corresponding author upon reasonable request.
