# Supplementary material for: Saliva as a non-invasive matrix for assessing xenobiotic metabolites and metabolomes: implications for maternal health and preeclampsia
Source: Int J Oral Sci. 2025 Jul 22;17:55. doi: 10.1038/s41368-025-00390-8 (PMC12283956; doi:10.1038/s41368-025-00390-8)
Supplement: Supplementary file 1 — Supplemental material [file 41368_2025_390_MOESM1_ESM.pdf]

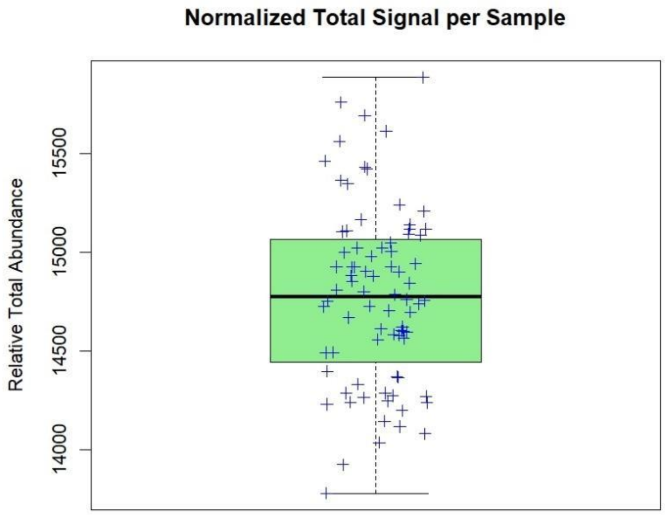

Figure S1. Box plot showing samples within the IQR range (Lower whisker = Q1 − 1.5 × IQR and Upper whisker = Q3 + 1.5 × IQR)

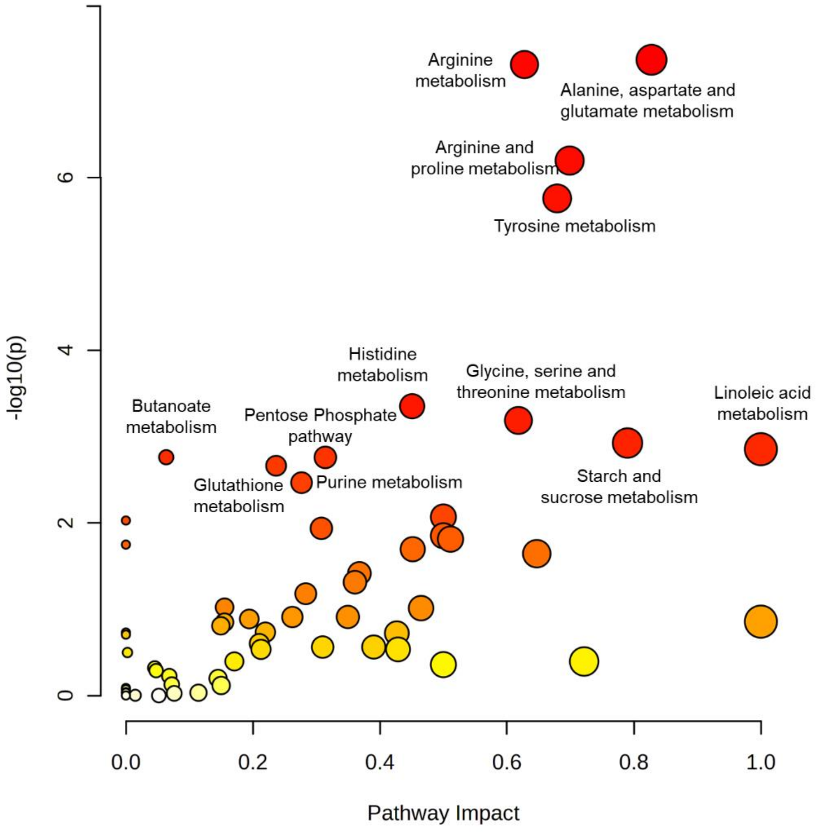

Figure S2. Pathway analysis of identified salivary metabolites using the KEGG Pathway Database. Each circle represents a metabolic pathway, with the x-axis indicating the *pathway impact* based on pathway topology analysis and the y-axis showing the statistical significance of enrichment as $-\log_{10}(p)$ values. Larger and darker red circles indicate more significant and impactful pathways.

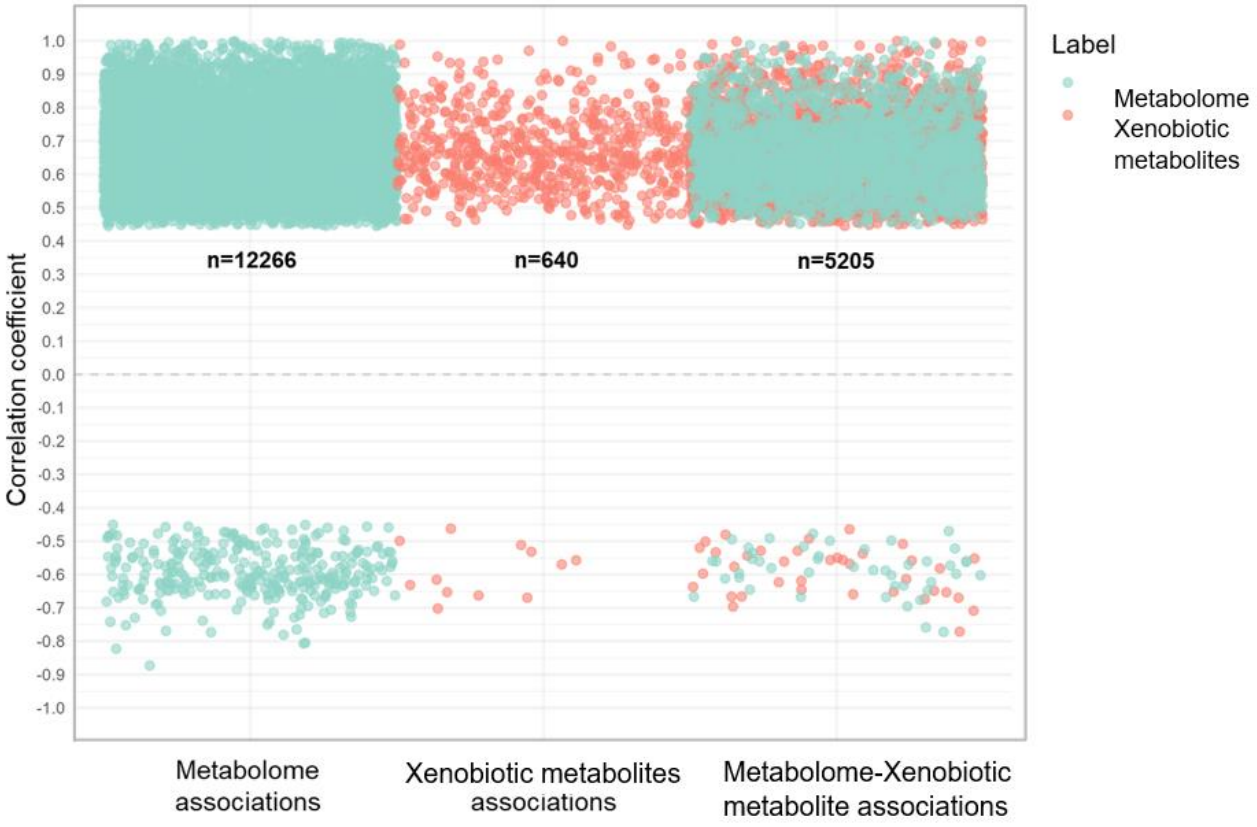

Figure S3. Miami Plot depicting the association between endogenous metabolites and xenobiotic metabolites (n=18,109). Each point corresponds to a metabolome/xenobiotic metabolite association obtained using Pearson correlation analysis; the y-axes show the correlation coefficient, and the x-axis represents the association within and between groups.
